# Release of soluble vascular endothelial growth factor receptor-1 (sFlt-1) during coronary artery bypass surgery

**DOI:** 10.1186/1749-8090-2-38

**Published:** 2007-09-21

**Authors:** Yves Denizot, Alexandre Leguyader, Elisabeth Cornu, Marc Laskar, Isabelle Orsel, Christelle Vincent, Nathalie Nathan

**Affiliations:** 1 UMR CNRS 6101, Centre National de la Recherche Scientifique, Université de Limoges, France; 2 Service de Chirurgie Thoracique et Cardiovasculaire, CHU Dupuytren, Limoges, France; 3 Service d'Anesthésie Réanimation Chirurgicale, CHU Dupuytren, Limoges, France

## Abstract

**Background:**

This study was conducted to follow plasma concentrations of sFlt-1 and sKDR, two soluble forms of the vascular endothelial growth factor (VEGF) receptor in patients undergoing coronary artery bypass graft (CABG) surgery with extracorporeal circulation (ECC).

**Methods:**

Plasma samples were obtained before, during and after surgery in 15 patients scheduled to undergo CABG. Levels of sFlt-1 and KDR levels were investigated using specific ELISA.

**Results:**

A 75-fold increase of sFlt-1 was found during cardiac surgery, sFlt-1 levels returning to pre-operative values at the 6^th ^post-operative hour. In contrast sKDR levels did not change during surgery. The ECC-derived sFlt-1 was functional as judge by its inhibitory effect on the VEGF mitogenic response in human umbilical vein endothelial cells (HUVECs). Kinetic experiments revealed sFlt-1 release immediately after the beginning of ECC suggesting a proteolysis of its membrane form (mFlt-1) rather than an elevated transcription/translation process. Flow cytometry analysis highlighted no effect of ECC on the shedding of mFlt-1 on platelets and leukocytes suggesting vascular endothelial cell as a putative cell source for the ECC-derived sFlt-1.

**Conclusion:**

sFlt-1 is released during CABG with ECC. It might be suggested that sFlt-1 production, by neutralizing VEGF and/or by inactivating membrane-bound Flt-1 and KDR receptors, might play a role in the occurrence of post-CABG complication.

## Introduction

Coronary artery bypass graft (CABG) surgery with extracorporeal circulation (ECC) is associated with an inflammatory response because, among numerous other causes, of blood contact with the artificial bypass surface, cold cardiac ischaemia and hypothermia [1-3]. Various studies have highlighted alterations in lipidic, cytokine and haematopoietic colony stimulating factor (CSF) networks during and after CABG surgery [4-10]. Thus, circulating levels as platelet-activating factor, leukotriene B_4_, thromboxane B_2_, interleukin (IL)-6, IL-8, IL-10, soluble IL-1 receptors, soluble tumour necrosis factor alpha (TNF-α) receptors, macrophage-CSF (M-CSF) and granulocyte-CSF (G-CSF) are altered during and after surgery and might be involved in the post-CABG multiple organ failure syndrome. It is now clear that these productions did not reflect an unspecific inflammatory state since levels of IL-4, IL-13, leukemia inhibitory factor, GM-CSF, and soluble IL-6 receptors remain unchanged during and after cardiac surgery [6,8,9].

The angiogenic network is also affected during and after CABG [10]. Among angiogenic growth factors, vascular endothelial growth factor (VEGF) fulfils a central role in the formation and function of blood vessels and during vascular healing in response, for example, to vascular trauma induced by mechanical disruption [11,12]. VEGF has been characterized as a heparin binding angiogenic growth factor displaying high specificity for endothelial cells. The expression of VEGF is stimulated in response to hypoxia and by a wide range of inflammatory cytokines. In vivo VEGF induces angiogenesis as well as permeabilisation of blood vessels and play central role in the regulation of vasculogenesis [13]. VEGF receptor (VEGFR) family consists of three members Flt-1 (VEGFR-1), KDR (VEGFR-2) and Flt-4 (VEGFR-3), all of which belong to the receptor tyrosine kinase superfamily [13]. Flt-1 and KDR exhibited high affinity for VEGF. Flt-4 is closely related in structure to the products of the Flt-1 and KDR genes. However, VEGF did not show specific binding for Flt-4 and its expression is restricted to developing lymphatic vessels. Soluble forms of the Flt-1 (sFlt-1) and KDR (sKDR) are found in human plasma [13]. Studies highlighted that soluble form of receptors are capable of sequestering ligand and preventing signal transduction. Excessive placental sFlt-1 production, by neutralizing VEGF, may play a causal role in the pathogenesis of the maternal preeclampsia [14]. Studies reported an association between coronary artery disease or myocardial infarction and elevated circulating levels of VEGF [15,16]. Of interest, reduced circulating levels of sFlt-1 were found in these patients suggesting lost of the endogenous compensatory anti-inflammatory mechanism induced by sFlt-1. Strengthening this hypothesis, sFlt-1 was recently reported to attenuate sepsis morbidity and mortality in an experimental mouse model by improving both cardiac and lung functions as compared with untreated animals [17]. Whether circulating VEGF levels are affected after CABG [10,18,19], no data are currently available concerning values of its soluble receptors. This study was conducted to elucidate whether sFlt-1 and sKDR were released during CABG with ECC.

## Patients and methods

The investigation conforms with the principles outlined in the Declaration of Helsinki. Fifteen patients scheduled to undergo CABG were included in this study. All patients had a preoperative ejection fraction above 40%. Anaesthesia was induced and maintained with titrated doses of fentanyl and flunitrazepam. Muscular relaxation was achieved with pancuronium (0.1 mg/kg). Membrane oxygenators were used. The blood was harvested from the surgical field and from the cell saver at the end of ECC and reinfused to all patients. All patients received high doses of aprotinin. Plasma samples were collected from the radial artery catheter before vascular cannulation and after opening the chest (T_0_), at the end of ECC just before (T_1_) and after cross clamp release (T_2_), after weaning from ECC (T_3_), at the 6^th ^(T_4_) and 24^th ^post-operative hour (T_5_). All patients had uneventful surgery. Plasma samples were collected between January 1995 and December 1996 [4] and were stored at -80°C until sFlt-1 and sKDR assays.

In another set of experiments, an in order to test more precisely the kinetic of sFlt-1 release, blood samples were harvested from a new set of 10 patients before ECC (T_0_), immediately at the beginning of ECC before aortic cross clamp (T_1'_) or after aortic cross clamp (T_2'_). Leukocytes and platelets were counted in a haemocytometer. Blood mononuclear cells were recovered on a Ficoll gradient in order to extract their RNA contents. Aliquots of blood were immediately used for flow cytometry analysis of membrane Flt-1 (mFlt-1) on platelets, monocytes, lymphocytes and granulocytes. Plasma samples were stored at -80°C until sFlt-1 and sKDR assays.

A decrease of plasma protein contents was observed during and after CABG surgery [1]. In order to avoid the influence of haemodilution during bypass, plasma sFlt-1 and sKDR levels were expressed by pg per mg of total plasma protein contents (pg/mg) measured at the simultaneous times. The proteinemia was determined by the BCA Protein Assay Reagent (Pierce, Rockford, IL). Plasma sFlt-1 and sKDR levels were measured with commercially ELISA kits (R&D Systems, Abingdon, UK). Biological results were expressed by pg/mg of protein measured at the simultaneous times. The sensitivity of the assays enables the detection of levels as low as 20 pg/ml and 30 pg/ml for sFlt-1 and sKDR, respectively. Results are expressed as mean ± SEM.

Blood mononuclear cells were separated on a Ficoll gradient (400 × g, 20 min) and washed two times with Hanks' balanced salts solution (HBSS). Total RNA was extracted with Tri Reagent^® ^(Ambion, Austin, TX). Reverse transcription (RT) was performed with 2.5 μg total RNA with the SuperScript reverse transcriptase (Invitrogen, Cergy Pontoise, France). Polymerase chain reaction (PCR) experiments for sFlt-1 transcripts were carried out with a sense primer 5'-TGTCAATGTGAAACCCCAGA-3' and an anti-sense primer 5'-GTCACACCTTGCTTCGG AAT-3' which amplify a 175 bp fragment of the human sFlt-1 gene. Amplification of glyceraldehyde-3-phosphate dehydrogenase (GAPDH) was performed to confirm the integrity of RNA. The GAPDH sense and anti-sense primers were 5'-GGCTGAGAACGGGAAGC TTG-3' and 5'-GGATGATGTTCTGGAGAGCC-3', amplifying a 439 bp fragment. cDNA was denatured 180 sec at 94°C, and then submitted to 40 cycles consisting in 94°C/60 sec, 58°c or 60°C (for sVEGFR-1 and GAPDH, respectively)/60 sec and 72°C/75 sec. Products were then analysed on an agarose gel stained with ethidium bromide. sFlt-1 PCR products were cloned in Topo™ cloning kit (Invitrogen) and sequenced by the dideoxynucleotide method on an ABI Prism 310 DNA genetic analyzer (Applied Biosystems, Foster City, CA).

The presence of mFlt-1 was tested by direct immunofluorescence staining of whole blood (acid-citrate-dextrose-anticoagulated) using fluorochrome-conjugated antibodies. Briefly 50 μl of whole blood were incubated with 20 μl of mouse IgG_1 _anti-human Flt-1 conjugated with phycoerythrin (PE) or irrelevant mouse IgG_1 _PE-conjugated isotype control (R&D systems, Minneapolis, MN) for 30 min at 4°C. Red blood cells were lysed, washed in PBS buffer, and samples were incubated with anti-CD45 PC7 labelled antibodies. After washing, cells were then submitted to flow cytometric analysis (XL II, Coulter, Margency, France). Platelets, lymphocytes, monocytes and granulocytes were identified based on the typical morphology in the forward scatter/side scatter cytogram and with the CD45 labelling.

Human umbilical vein endothelial cells (HUVECs) were purchased from Promo Cell (Heidelberg, Germany). HUVECs were grown in the media provided by the vendor and cultures were routinely used between passages 4 and 7. HUVECs (2 × 10^3 ^cells) in 100 μl of growth media were plated into 96-well plates and cultured for 3 days at 37°C in 5% CO_2 _in air. HUVECs were cultured in the presence or absence of 10 ng VEGF (PeproTech, London, UK), 10 μl of plasma collected during ECC (T_2_) and/or 0.25 μg rabbit anti-soluble Flt-1 (Zymed Laboratories, San Francisco, CA). Cell proliferation (in sixplicates) was measured by a colorimetric method using MTS (Promega, Madison, WI). The MTS tetrazolium compound is bioreduced by cells into a colored formazan product that is soluble in tissue culture medium. This conversion is accomplished by NADPH or NADH produced by dehydrogenase enzymes in metabolically active cells. In some experiments the number of cultured cells (in quadruplicate) was directly evaluated using a haemocytometer.

Results are expressed as mean ± SEM. Statistical analysis was done using Mann-Whitney U-test. A p < 0.05 was considered significant.

## Results

**Figure 1:**
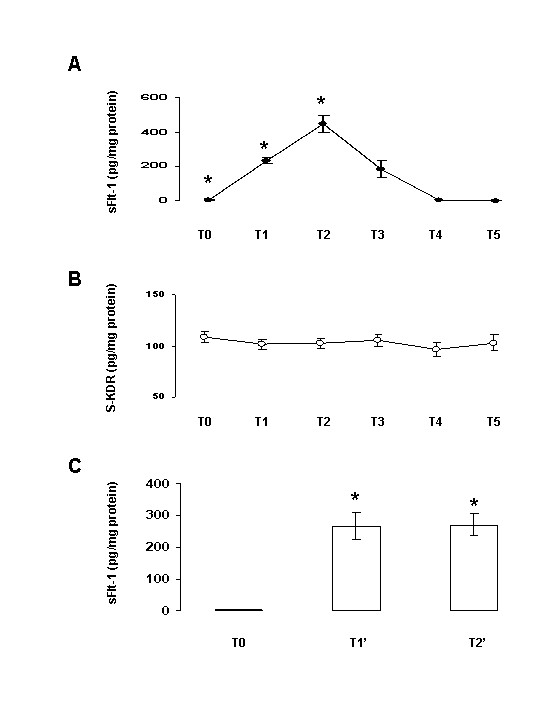
**Plasma sFlt1 and sKDR levels during and after cardiopulmonary bypass graft surgery**. A: Plasma sFlt-1 values are expressed in pg per mg protein. T0: before vascular cannulation and after opening the chest; T_1_: during extracorporeal circulation (ECC); T_2_: at the end of ECC before cross clamp release; T_3_: after cross clamp release; T4: the 6th post-operative hour; T5: the 24th post-operative hour. Mean ± SEM of 15 patients. *p < 0.001 as compared with T0 (Mann-Whitney U-test). B: Plasma sKDR levels are expressed in pg/mg protein. Same blood sampling time than in A. Mean ± SEM of 15 patients. *p < 0.001 as compared with T0 (Mann-Whitney U-test). No statistical differences were observed (Mann-Whitney U-test). C: Plasma sFlt-1 values at the beginning of ECC. T0: before vascular cannulation and after opening the chest; T_1'_: before cross clamp; T_2'_: after cross clamp. Mean ± SEM of 10 patients. *p = 0.0002 as compared with T0 (Mann-Whitney U-test).

Initial concentrations of plasma VEGF of these patients were evaluated at 3 pg/mg [10]. As shown in Figure 1A, a 75-fold increase of plasma sFlt-1 concentrations was found during CABG surgery. Levels increased significantly (p = 0.0001) during ECC (234.2 ± 16.2 pg/mg), reached maximum values at the end of ECC before cross clamp release (446.0 ± 49.3 pg/mg) and returned to pre-operative values (5.9 ± 2.7 pg/mg) at the 6^th ^post-operative hour (5.1 ± 0.8 pg/mg). Raw data of plasma sFlt-1 levels were 301.6 ± 135.6 pg/ml and 14915.0 ± 1629.2 pg/ml before ECC (T_0_) and at the end of ECC (T_2_), respectively. In contrast to sFlt-1, plasma sKDR levels did not significantly (p > 0.2) change during and after CABG surgery (Fig. 1B). As shown in Figure 1C, sFlt-1 levels significantly (p = 0.0002) increased immediately after the beginning of ECC.

**Figure 2:**
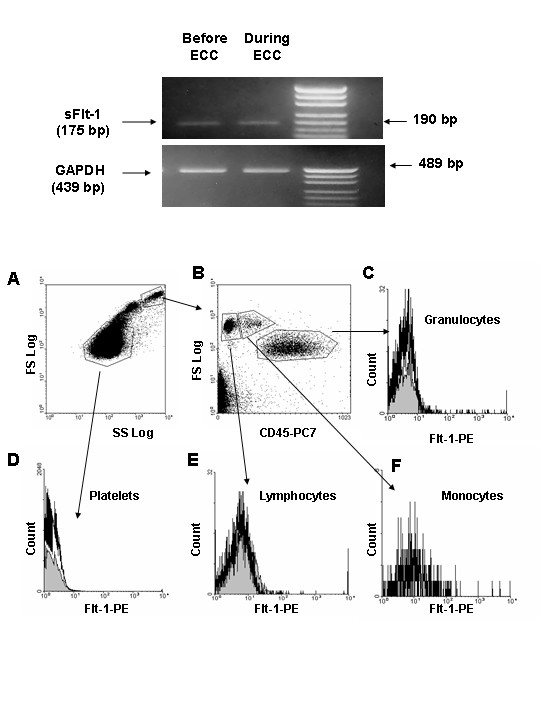
**RT-PCR analysis of sFlt-1 transcripts and mFlt-1 on blood mononuclear cells before and during ECC**. A: PCR experiments were carried out to amplify a 175 bp fragment of the human sFlt-1 receptor. GAPDH amplification (a 439 bp fragment) was performed to highlight the integrity of blood mononuclear cell mRNA. PCR products were analysed on a 1.2% agarose gel. Sizes of PCR products and DNA ladder are indicated by arrows. One representative experiment out of three is shown. B: Blood samples were collected before and during ECC. Forward scatter (FS) and side scatter (SS) were used to identify platelets and leukocytes (A). CD45 expression was used to separate lymphocytes, monocytes and granulocytes (B). Flow cytometry revealed no significant effect of ECC on mFlt-1 expression on platelets (C), lymphocytes (D), monocytes (E) and granulmocytes (F). The solid line represents blood cells before ECC staining with Flt-1-PE antibodies. The shaded area represents blood cells during ECC staining with Flt-1-PE antibodies. One experiment representative of 3 is shown.

We then investigated if white blood cells might be putative cellular sources for the ECC-derived sFlt-1 synthesis. In a first set of experiments sFlt-1 transcripts were evaluated in blood mononuclear cells of patients before and during ECC. PCR experiments did not reveal differences for sFlt-1 transcript levels before and after initiation of ECC (Figure 2, upper panel). The release of sFlt-1 was recently reported through proteolysis of its membrane form [20]. In a second set of experiments we, thus, evaluated the effect of ECC on mFlt-1 expression on circulating blood cell types (*i.e; *monocytes, lymphocytes, granulocytes and platelets). As shown in Figure 2A (lower panel), forward scatter and side scatter were used to identify platelets and leukocytes. Figure 2B illustrates the gates used to characterize lymphocytes, monocytes and granulocytes among CD45^+ ^cells. ECC had no effect on the basal expression of mFlt-1 on granulocytes, platelets, lymphocytes and monocytes (Figures 2C, 2D, 2E and 2F, respectively).

**Figure 3:**
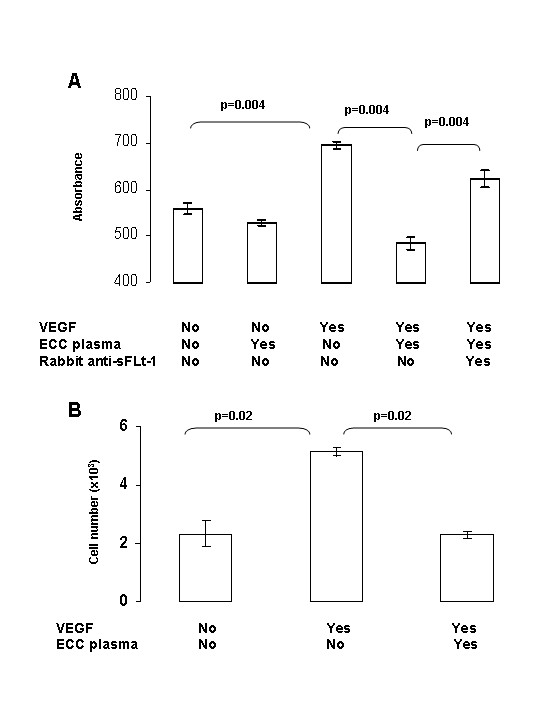
**Effect of ECC-derived sFlt-1 on HUVEC proliferation**. HUVECs (2 × 10^3 ^cells) were cultured alone or in the presence of 10 ng VEGF, 10 μl of plasma collected at T_2 _during ECC and/or 0.25 μg rabbit anti-soluble Flt-1. A: Cell proliferation (in sixplicates) was measured by a colorimetric method. One representative experiment out of 3 is shown. B: Cell number (in quadruplicate) was determined by direct cell counts. One representative experiment out of 2 is shown. Statistical differences were made using the Mann-Whitney U-test.

sFlt-1 was released during ECC. We then investigated the activity of the ECC-derived sFlt-1 on the VEGF mitogenic response in HUVECs by using a colorimetric method. As shown in Figure 3A, ECC-derived sFlt-1 markedly inhibited the VEGF mitogenic response in HUVECs; the effect being abolished by the use of anti-sFlt-1 antibodies. Cell counts (Figure 3B) confirmed the effect of the ECC-derived sFlt-1 on the growth of VEGF-stimulated HUVECs.

## Discussion

VEGF was first characterized as a potent endothelial growth factor and stimulator of endothelial permeability. Studies highlighted that sFlt-1 is capable of sequestering VEGF and preventing signal transduction [13]. Moreover, sFlt-1 can act as an endogenous VEGF inhibitor not only by sequestering VEGF but also by binding and inactivating membrane-bound Flt-1 and KDR receptors through a mechanism involving receptor homodimerisation or heterodimerisation [21]. The presence of sFlt-1 in serum and plasma of healthy donors strongly suggest that sFlt-1 might have an important function in the fine regulation of VEGF mediated activities in vivo. Beside its effect on endothelial permeability, VEGF is also reported to exhibit several pro-inflammatory and procoagulant activities [22-24]. Data have demonstrated elevated VEGF levels following CABG surgery [10,18,19], suggesting that VEGF is a component of the inflammatory network after open heart surgery. In this study we showed a dramatic (up to 75-fold) increase of circulating sFlt-1 levels during ECC. In contrast sKDR levels were unchanged strengthening the idea that sFlt-1 release is not only the consequence of an unspecific release in response to cardiac surgery. The ECC-derived sFlt-1 is functional as judged by its effect in the VEGF-induced proliferation of HUVECs. The cell source and the mechanism of these elevated levels of sFlt-1 remain speculative. During the past decade sFlt-1 was reported to be generated by an alternative splicing of the mFlt-1 transcript [13]. In our study, sFlt-1 levels peaked during ECC returning to pre-operative levels at the 6^th ^post operative hours. This rapid kinetics of production does not fit well with an elevated transcription, translation and/or maturation process. Two hypothesises might, thus, be suggested. The first one is the presence of pre-existing intracellular pool of sFlt-1 that might be released upon cell stimulation. The presence of intracellular pool of soluble Toll-like receptors and tumour necrosis factor alpha (TNF-α) receptors have been already described [25,26]. Of interest, the presence of KDR intracellular pools have been recently reported in endothelial cells [27], suggesting the putative existence of similar Flt-1 ones. The second hypothesis is a proteolysis process. Numerous transmembrane cell surface proteins exist as soluble forms suggesting that their cleavage and release from the cell surface represent a rapid and efficient post-translational regulatory mechanism. The proteolytic solubilisation of the ligand-binding cytokine receptor ectodomains not only renders cells insensitive toward cytokine effects. The capacity of soluble receptor forms to bind their ligands may prevent the cytokine binding to cell-bound receptors. Soluble receptors for leukocyte and endothelial cell activation molecules (Fas ligand, CD40, CD93), for cytokine receptors (IL-1, IL-2, IL-6, TNF-α), for adhesion and locomotion molecules (CD44, CXCL16, ICAM-1, VCAM-1), for cell-to-cell adhesion molecules involved in endothelial diapedesis (PECAM-1, VE-cadherin), and for growth factors (bFGF, PDGF) are released by proteolysis by a wide array of enzymatic activities including neutrophil-derived serine proteases, secretases (sheddases), metalloproteases and ADAMs [28-30]. In our study, flow cytometry analysis did not highlight any effect of ECC on mFlt-1 levels on white blood cells and platelets. Recently a γ-secretase-dependent Flt-1 intramembrane proteolysis was found in vascular endothelial cells reporting, for the first time, a shedding contribution for the sFlt-1 receptor [20]. The ectodomain shedding of mFlt-1 on vascular endothelial cells might, thus, be a putative mechanism explaining the rapid release of sFlt-1 in blood during ECC. Of interest serine proteases, metalloproteases and ADAM are activated during cardiopulmonary bypass [31-33].

## Conclusion

sFlt-1 is released during CABG with ECC. The precise mechanisms of this sFlt-1 production remain to be elucidated. The existence of pre-existing intracellular endothelial pool of sFlt-1 that might be released upon cell stimulation or the shedding of endothelial mFlt-1 may be two putative mechanisms. At this time the physiologic meaning of these elevated sFlt-1 levels remains speculative. It might be suggested that sFlt-1 production, by neutralizing VEGF and/or by inactivating membrane-bound Flt-1 and KDR receptors, might play a role in the occurrence of post-CABG complications. Investigation of sFlt-1 levels in patients with post-CABG complications would be of interest to test this hypothesis. If the release of sFlt-1 is benefit to reduce post-CABG complications sFlt-1 might became a potential target for preoperative intervention in the form of sFlt-1 pharmacologic therapy to prevent or ameliorate some postoperative complications of CABG by interfering very early in the VEGF-induced inflammatory cascade rather than later when the number of proinflammatory molecules and their associated damages are greater. Animal models have still highlighted the interest of sFlt-1 administration in order to reduce disease severity in experimental inflammatory diseases and sepsis [17,34,35]. Finally, the activation of VEGFR-1 is essential for recruitment of adult stem cells. Endothelial progenitors are mobilized into the circulation after CABG [36]. During CABG it is possible that the increase concentration of sFlt-1 could affect the recruitment of endothelial cell progenitors with adverse effects on cardiac physiology after bypass procedure. Strengthening this hypothesis decrease and dysfunction of endothelial progenitor cells have been found in umbilical cord blood with maternal pre-eclampsia; these alterations being associated with the increased sFlt-1 levels in the umbilical cord blood [37].

## Abbreviations

CABG – coronary artery bypass graft, ECC – extracorporeal circulation, HUVECs – human umbilical vein endothelial cells, VEGF – vascular endothelial growth factor

## Competing interests

The author(s) declare that they have no competing interests.

## Authors' contributions

AL, EC, ML, IO and NN were the surgical and anaesthesia teams and carried out the preoperative clinical and analytical data collection, carried out blood samples and actively participate to the study design, data interpretation and manuscript writing.

YD and CV carried out the determination of sFlt-1 and sKDR values, RT-PCR experiments, flow cytometry analysis and interpretations and actively participate to manuscript writing.

All authors read and approved the final manuscript.
